# Axitinib: Newly Approved for Renal Cell Carcinoma

**DOI:** 10.6004/jadpro.2012.3.5.7

**Published:** 2012-09-01

**Authors:** Timothy Tyler

**Affiliations:** From Desert Regional Medical Center–Comprehensive Cancer Center, Palm Springs, California

Data presented at the June 2012 American Society of Clinical Oncology (ASCO) annual meeting reaffirmed the use of axitinib (Inlyta), a potent and selective second-generation inhibitor of vascular endothelial growth factor receptors, for second-line treatment of metastatic renal cell carcinoma (mRCC). Viable treatment options have rapidly progressed from literally almost nothing in the way of effective chemotherapy or targeted therapies less than a decade ago to having to choose from seven new US Food and Drug Administration (FDA)-approved therapies today. The addition of yet another therapy can make it challenging for oncology advanced practitioners (APs) to stay current with respect to the many developments in renal cancer. This article will seek to provide APs with some insight into this most recent drug approval.

## Background

The most recent data from the American Cancer Society (ACS) show renal cell carcinoma trending at about 65,000 new cases per year in the United States, with the death toll well over 13,000 annually (favoring males 2:1). Although the numbers seem to be improving, they are already not quite as grim as in other cancers, with a 70% 5-year survival for kidney cancer at present (Siegel, Naishadham, & Jemal, 2012). This rate is somewhat skewed by high survival for patients with local disease vs. the lower survival rates for disease in the renal pelvis. Surgery remains the definitive treatment of choice for kidney cancer where possible; historically, chemotherapy has not offered much improvement prior to the addition of more targeted therapies to the treatment armamentarium. This has stepped up dramatically since 2006 in that there are now newer therapies (see Table 1) that are considered more targeted and distinct from traditional chemotherapy (Grünwald & Merseburger, 2012).

**Table 1:**
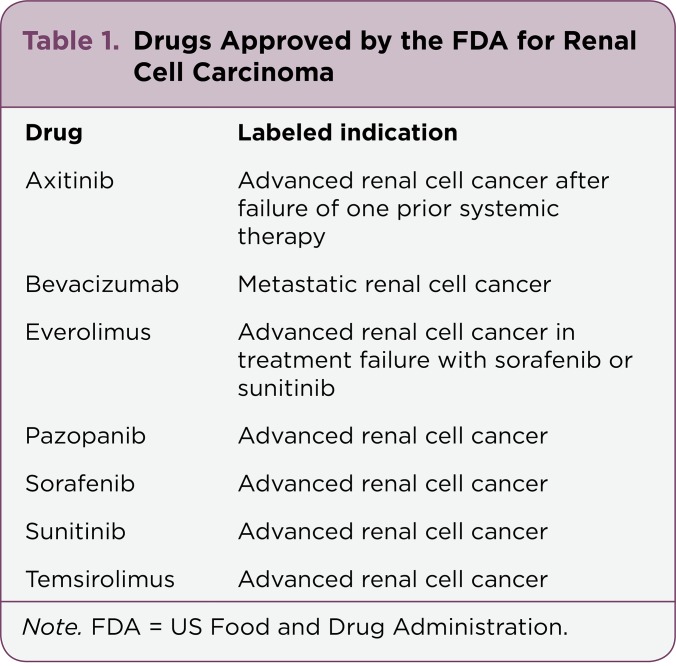
Table 1. Drugs Approved by the FDA for Renal Cell Carcinoma

Axitinib was recommended for FDA approval by the Oncology Drug Advisory Committee (ODAC) in late 2011; full approval was granted on January 27, 2012. The approved indication is *"…for the treatment of advanced renal cell carcinoma [RCC] after failure of one prior systemic therapy"* (Pfizer, 2012). Since axitinib’s approval to treat advanced RCC in the second-line setting, more compelling data demonstrating its role in the first-line setting were presented at the 2012 ASCO annual meeting; however, the FDA’s approval is currently only for second-line therapy after the failure of one prior therapy.

## Trial Results

First-line data presented at the 2012 ASCO annual meeting did demonstrate an improvement, with greater than 1-year median progression-free survival (mPFS) and overall response rate (ORR) ranging from 40% to 56% (Rini et al., 2012). While these are compelling data, the results did demonstrate that the limiting toxicity of this agent will be hypertension. A second presentation also seemed to indicate that a rise in blood pressure may actually be a marker of response if seen within the first 2 weeks in the trial setting; this has been hypothesized as related to the area under the curve (AUC) of the medication. Patients who had drug exposure above the therapeutic threshold on cycle 1, day 15, had longer mPFS and higher ORR vs. the patients with subtherapeutic exposure. Use of this agent in the first-line setting will require further study, as the drug has demonstrated definitive activity with its second-line indication (Rini et al., 2012; Michaelson et al., 2012).

In the advanced disease setting there was report of fatigue, diarrhea, and palmar-plantar erythrodysesthesia (hand-foot syndrome), but this was in a heavily pretreated population. The question of whether these side effects will remain as pronounced in a first-line setting is still unanswered. Of equal importance is whether or not the 2-month survival advantage will stay steady, grow, or shrink. For the oncology advanced practitioner, these questions, as well as how to best tailor therapy to patients’ unique parameters, make management of RCC almost as challenging as when there were too few choices available (Rini et al., 2011).

## Dosing and Administration

Axitinib comes in 1-mg and 5-mg tablets and is dosed twice daily with the starting dose recommended at 5 mg dosed approximately 12 hours apart daily with no regard to meals; however, patients should be instructed to take the medication with an entire glass of water. Dose adjustments are to be made upon patient response to safety and tolerability. Patients being initiated on axitinib should have well-controlled blood pressure, as hypertension was identified as an issue during the clinical trials. For patients that have resistant hypertension even in the face of antihypertensive medication management, the manufacturer recommends a dose reduction of axitinib. Fatalities were seen in the clinical trials with hemorrhage and thrombotic events, so caution should be exercised in patients at risk for thrombotic events and in those with bleeding problems (Pfizer, 2012).

Dose escalation is possible over the course of treatment, but only for those patients tolerating the drug for 2 weeks with no toxicity greater than grade 2 who are not hypertensive and who do not require medication management for hypertension. When increasing the dosing, the first dose escalation is recommended to 7 mg twice daily; the next escalation would be to 10 mg twice daily again after another 2-week trial with no toxicity over grade 2 (Pfizer, 2012).

The concomitant use of strong CYP3A4/5 inhibitors such as -azole antifungals and antiretrovirals is discouraged while the patient is on axitinib therapy (see Table 2). In the event that this is not possible, the manufacturer recommends the dose of axitinib be reduced by one half. The same dosing reduction by one half is suggested in the package insert as a starting dose for patients with baseline moderate hepatic impairment (Child-Pugh class B). If the concomitant use of a strong CYP3A4/5 inducer, such as many of the antiseizure medications, is unavoidable, the manufacturer offers no dosing advice other than to state that the plasma drug concentration of axitinib would likely be lower than therapeutic (Pfizer, 2012).

**Table 2:**
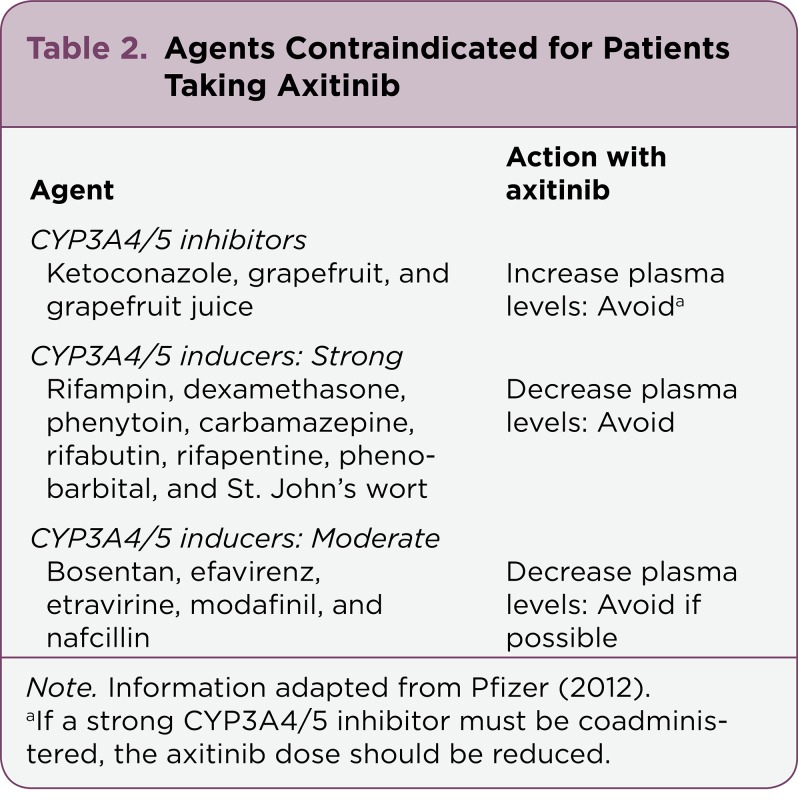
Table 2. Agents Contraindicated for Patients Taking Axitinib

## Side Effects and Cost

The major side effects that can be expected with axitinib (more than 20% of the time) are diarrhea or constipation, hypertension, fatigue, dysphonia, decreased appetite with weight loss, nausea, vomiting, and hand-foot syndrome. Other points to consider are the need to hold the medication 24 hours prior to surgery and to monitor for proteinuria before initiation and periodically during treatment. If moderate to severe proteinuria develops, the recommendation is to reduce the dose or even temporarily interrupt treatment (Pfizer, 2012; Elsevier Gold Standard, 2012).

Axitinib, which is currently listed at an average wholesale price of $58.80 per 1-mg tablet (McKesson Connect-Rx, 2012), is available through specialty pharmacies. Clinicians can visit www.pfizerpro.com for information about specific specialty pharmacies as well as patient assistance, or access the page directly by scanning the barcode on page 334 with a smartphone or tablet.

## Conclusion

In the renal cancer setting, oncology APs must stay abreast of several therapeutic options available for their patients. With the recent FDA approval of axitinib, oncology APs have yet another viable option for patients who have failed to respond to one previous therapy. This most recent addition to the renal cell cancer armamentarium is currently being studied in the first-line setting for mRCC and explored in other solid tumors.
